# Metabolic hubs under attack: Effector-mediated manipulation of plant inositol phosphate signaling

**DOI:** 10.1371/journal.ppat.1013649

**Published:** 2025-10-31

**Authors:** Elena Roitsch, Gabriel Schaaf, Thomas Lahaye, Martina K. Ried-Lasi

**Affiliations:** 1 Symbiosis Signalling Group, Department of Molecular Signal Processing, Leibniz Institute of Plant Biochemistry, Halle (Saale), Germany; 2 University of Bonn, Institute of Crop Science and Resource Conservation (INRES), Bonn, Germany; 3 University of Tübingen, ZMBP—General Genetics, Tuebingen, Germany; University of Tübingen: Eberhard Karls Universitat Tubingen, GERMANY

## Microbial effectors and their host targets: From proteins to metabolites

Microbial plant pathogens, including bacteria and fungi, deliver effector proteins into host cells to promote susceptibility—a phenomenon known as effector-triggered susceptibility (ETS, [1]). In response, host plants have evolved resistance (R) proteins that recognize specific effectors and execute effector-triggered immunity (ETI, [2]), which often culminates in a hypersensitive cell death (HR) response [1].

R proteins differ in the mechanistic basis by which they perceive effectors. Some directly bind their cognate effector, a concept known as the receptor-ligand model. Other R proteins sense the effector’s impact on its host targets [3], a strategy known as the guard model. Recent structural studies of R protein complexes have revealed small metabolites as indispensable elements of R protein-orchestrated immune responses [4]. It therefore seems plausible that microbial effectors target not only host proteins but also regulatory metabolites to suppress plant immunity—a hypothesis that remains to be experimentally validated.

To investigate how microbial effectors promote disease or activate R proteins, researchers commonly use effector-omics approaches such as yeast two-hybrid, co-immunoprecipitation, and proximity labeling to identify proteinaceous effector targets. Notably, these approaches inherently exclude non-protein molecules, introducing a bias in our understanding of effector–host interactions. High-throughput metabolomic techniques, such as untargeted LC–MS/MS profiling, can be used to systematically monitor metabolic changes upon activation of disease resistance factors. In addition, methods such as stable isotope tracing, metabolic flux analyses, or affinity-based capture of metabolites can help identify potential small-molecule targets or pathways affected by immune activation. Incorporating such approaches will be important to uncover the currently underexplored role of metabolites in plant immunity.

Recent studies have revealed that microbial effectors can indeed target metabolites, particularly inositol phosphates (InsPs), which are key signaling molecules at the interface of nutrient sensing and immune regulation [5,6]. The biosynthesis and physiological functions of InsPs and their pyrophosphorylated derivatives (inositol pyrophosphates, PP-InsPs) have been comprehensively reviewed [5], highlighting their central role in coordinating plant responses to abiotic and biotic stress. This Pearls article explores the emerging view that InsP signaling represents a conserved and strategic target exploited by microbial effectors to reprogram host cellular processes [7–10].

## Inositol phosphates: An emerging target of microbial effectors

InsPs serve as key integrators of metabolic status, environmental cues, and hormonal signals in plant cells, making them attractive targets for microbial effectors. InsPs consist of a *myo*-inositol ring, a cyclic alcohol with six C atoms, which theoretically can be modified with multiple (pyro)phosphate groups, giving rise to a vast variety of InsP species For example, InsP_6_, also known as phytate, is the fully phosphorylated form of *myo*-inositol, carrying one phosphoryl group (hereafter referred to as phosphate group) at each of the six carbon positions. The PP-InsP 5-InsP_7_ is generated from InsP_6_ by the addition of a phosphate group to the already phosphorylated position 5 of the *myo*-inositol ring, creating a high-energy pyrophosphate moiety. These modifications result in molecular structures with unique configurations of mono- and pyrophosphorylated positions that serve as specific “keys,” fitting into corresponding “locks” to either activate or suppress specific downstream responses. Despite the diversity of theoretically possible InsPs, only a subset of these potential “keys” has been detected and characterized in plants, and many of their corresponding “locks,” that is, the biological processes they regulate, remain unidentified.

Previous studies suggest that PP-InsPs might be able to act in spatially restricted microenvironments. For example, Laha and colleagues (2022) reported a physical interaction of the InsP_6_ kinase ITPK1 with the F-box component Transport Inhibitor Response 1 (TIR1; [11]) of the auxin receptor complex [12], suggesting that close proximity between biosynthetic enzymes and effector complexes may generate a localized enrichment of 5-InsP_7_ regulating auxin signaling. This concept is similar to that proposed for the sequence-unrelated InsP_6_ kinase IP6K2, whose interaction with the Tti1/Tel2/Tti2 (TTT) complex potentiates casein kinase 2-dependent regulation of this complex in mammalian cells [13]. Such localized signaling may be more relevant than global abundance in determining specificity.

Attention has focused on 5-InsP_7_ and InsP_8_, which regulate phosphate (P_i_) homeostasis by interacting with SPX-domain proteins that control the activity of PHR transcription factors [14–17]. Under Pi sufficient conditions, PP-InsPs stabilize SPX-PHR complexes, thereby repressing phosphate starvation responses (PSRs). Under Pi-deficient conditions, declining PP-InsP levels release PHR transcription factors from SPX-mediated repression, leading to PHR-dependent expressional changes. Beyond Pi homeostasis, InsPs also participate in regulation of hormone signaling and plant immunity [10,12,18–22], as well as nitrate sensing [23]. Notably, these regulatory networks are likely interconnected, rather than independently regulated by distinct InsP species.

By manipulating InsPs or InsP metabolism, microbial pathogens may mimic P_i_ starvation to promote nutrient acquisition and/or directly suppress immune responses. Although altered P_i_ signaling has been linked to immunity, the underlying causal relationships remain poorly understood. Deciphering the primary targets and precise mechanisms of how pathogen effectors modulate InsP signaling thus represents an exciting and understudied frontier in plant–microbe interactions.

## Bacterial effectors manipulating inositol phosphates: The case of XopH/AvrBs7

**Fig 1:**
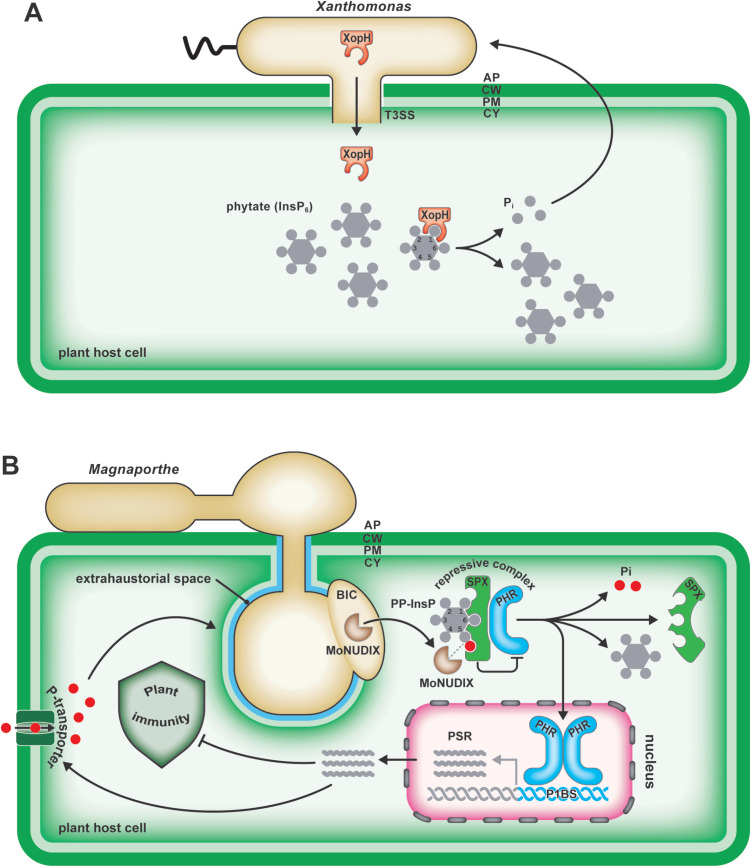
Microbial effectors dephosphorylate inositol phosphates to promote disease. **A)** The bacterial pathogen *Xanthomonas* uses a syringe-like injection system, known as the type III secretion system (T3SS), to deliver the effector XopH or orthologs as AvrBs7 into host cells. XopH dephosphorylates InsP_6_ (phytate) at the C1 position, classifying it as a 1-phytase. The released phosphate could increase intracellular and apoplastic phosphate (P_i_) levels, potentially promoting *Xanthomonas* growth *in planta*. CW (cell wall), PM (plasmamembrane), CY (cytoplasm), AP (apoplast). **B)** The fungal pathogen *Magnaporthe* translocates *Mo*NUDIX effectors into host cells, where they dephosphorylate inositol pyrophosphates (PP-InsPs). This dephosphorylation releases the transcription factor PHR from repressive SPX–PHR complexes, thereby activating the phosphate starvation response (PSR). The PSR is thought to promote microbial infection by suppressing plant immunity, enhancing phosphate availability for the pathogen, or both. BIC (biotrophic interfacial complexes).

Bacteria deliver effector proteins directly into plant host cells using a syringe-like apparatus known as the type III secretion system (T3SS) [24,25]. Among these T3 effectors (T3Es), AvrBs7 from the plant-pathogenic bacterium *Xanthomonas gardneri* and its ortholog XopH (formerly AvrBs1.1) from *X. euvesicatoria* exemplify bacterial effectors that directly target InsPs rather than conventional proteinaceous substrates (Fig 1A) [10,26].

Both effectors share high sequence similarity and trigger immune responses in pepper plants carrying the *R* gene *Bs7*, suggesting a conserved molecular function *in planta* [26]. Initially, XopH and AvrBs7 were annotated as protein phosphatases based on homology to known phosphatase domains and the presence of conserved catalytic residues [26]. *In vitro* assays confirmed phosphatase activity against synthetic peptide substrates, which was lost upon mutation of the catalytic cysteine residue [10,26]. Strikingly, the same mutants also abolished *Bs7*-mediated hypersensitive cell death *in planta*, linking enzymatic activity to effector recognition [10,26]. These observations initially supported the idea that XopH and AvrBs7 act as protein phosphatases within the host.

A conceptual breakthrough came with the discovery that both effectors are structurally related to bacterial phytases (Fig 1A) [10], enzymes that dephosphorylate phytate (*myo*-inositol-*hexakis*phosphate, InsP_6_), which serves as a phosphate storage molecule in plants [27]. XopH preferentially hydrolyzes InsP_6_ to InsP_5_ with up to 1,000-fold higher efficiency compared to the dephosphorylation of peptide substrates [10]. Remarkably, it cleaves specifically at the previously uncharacterized position 1 of the inositol ring, yielding the unique InsP_5_ [1-OH] isomer. This finding redefined the biologically relevant substrate of these effectors, establishing InsPs rather than proteins as their primary targets. Phytase activity of XopH/AvrBs7 is hypothesized to promote virulence through two non-mutually exclusive mechanisms: (i) by mobilizing P_i_ from host stores to support pathogen growth and/or (ii) by altering host immune signaling by depletion of InsP_6_ or the generation of signaling active InsP-derived metabolites (Fig 1A).

Taken together, these findings highlight a novel virulence strategy in which *Xanthomonas* spp. exploit inositol phosphate metabolism as a point of host manipulation, functionally linking metabolic interference with both infection success and immune activation (Fig 1A).

## Fungal effectors manipulating inositol phosphates: The case of NUDIX hydrolases

Unlike bacteria, which use the T3SS to inject effectors into host cells, fungal pathogens such as *Magnaporthe oryzae* invade the plant apoplast with hyphae and form specialized dome-like structures called biotrophic interfacial complexes (BICs) [28]. These act as effector reservoirs and translocation hubs, enabling the delivery of virulence proteins into the host cytoplasm [28]. High expression levels of fungal proteins in BICs often indicate their role as effectors that promote virulence/colonization of the plant host through their function inside the host cell, mirroring the intracellular function of bacterial T3Es.

In *M. oryzae*, the causative agent of blast disease in rice, wheat, and barley, the predicted effector protein *Mo*NUDIX is highly expressed in BICs during infection of rice plants (Fig 1B) [8], consistent with the idea that *Mo*NUDIX functions as a translocated effector in plant host cells. Genetic deletion of *MoNUDIX* significantly reduces hyphal growth and pathogenicity in both rice and barley, confirming its essential role in virulence [8]. *Mo*NUDIX shares homology with the NUDIX hydrolases, a class of enzymes that cleave diphospho-bonds of different nucleoside diphosphate derivatives [29] but also targets non-nucleoside pyrophosphate substrates [30–33]. In some cases, these enzymes exhibit specific activity toward PP-InsPs in yeast and mammals [32,34–36] and more recently, this activity has also been identified in plants [9,37–39].

Structural modeling of *Mo*NUDIX revealed a positively charged pocket characteristic of PP-InsP-binding sites, a feature conserved in related effectors from *Colletotrichum higginsianum* and *C. graminicola* [8]. While McCombe et al. demonstrated *Mo*NUDIX activity toward 5-InsP_7_ [8], work by Schneider et al. expanded on this by showing that *Mo*NUDIX and its homologs can also hydrolyze 4-, 6-, and, to a lesser extent, 1-InsP_7_, highlighting a broader substrate promiscuity [9].

Importantly, *Mo*NUDIX-dependent virulence is strictly linked to its enzymatic activity, paralleling the function of phytase-type bacterial effectors like XopH and AvrBs7 [8,10]. Host P_i_ status appears to modulate disease outcome, as elevated phosphate levels correlate with increased susceptibility to *M. oryzae* [40]. *Mo*NUDIX promotes phosphate starvation responses in host plants, likely facilitating the release of PHR transcription factors from inhibitory SPX complexes (Fig 1B) [8]. However, whether *Mo*NUDIX initiates P_i_ starvation responses as the primary virulence mechanism or whether virulence is achieved by another mechanism, and the depletion of InsP_7_ pools only leads to secondary activation of phosphate starvation responses remains an open question.

While InsP-targeting effectors have been clearly identified in bacteria and fungi, emerging evidence suggests similar mechanisms may exist in oomycetes. The *Phytophthora sojae* effector Avr3b carries a conserved NUDIX hydrolase motif and suppresses Avr1b-triggered ETI in *Nicotiana benthamiana* [41]. Although the conserved NUDIX motif of Avr3b is essential for its virulence function, it is dispensable for recognition by the soybean R protein Rps3b [41]. This contrasts with the bacterial effectors XopH and AvrBs7 where mutations in the predicted catalytic sites abolish recognition by the corresponding pepper R protein Bs7 [26]. Avr3b is structurally related to *Arabidopsis thaliana* NUDT7, a known ADP-ribose/NADH pyrophosphatases that negatively regulates resistance against both the bacterial pathogen *Pseudomonas syringae* and the obligate biotrophic oomycete pathogen *Hyaloperonospora arabidopsidis* [42]. Initial studies suggested that Avr3b might mimic such host regulators to suppress defence responses via NADH and ADP-ribose hydrolysis [41]. However, more recent biochemical analysis showed that recombinant Avr3b expressed in *Escherichia coli* lacks NADH hydrolase activity [43].

Intriguingly, Avr3b shows high sequence similarity with yeast *Sc*DDP1, a NUDIX hydrolase with defined activity on PP-InsPs, particularly 1-InsP_7_ and InsP_8_ [44]. This raises the possibility that Avr3b, like AvrBs7, XopH, and *Mo*NUDIX and its homologs, may target PP-InsPs *in planta*. Although this remains to be experimentally demonstrated, it opens a compelling avenue for further research into InsP-targeting effectors in oomycetes, a group so far underrepresented in this context.

## Inositol phosphates as central nodes in plant–pathogen interactions

The independent evolution of effectors capable of targeting InsPs across bacteria, fungi, and potentially also oomycetes underscores the strategic significance of InsP signaling as a conserved metabolic interface exploited by microbial pathogens. By mimicking host enzymes or intercepting key signaling intermediates, pathogens appear to co-opt host InsP/PP-InsP metabolism to rewire host nutrient sensing and/or immune regulation.

While the enzymatic degradation of InsPs by microbial hydrolases is now clearly linked to virulence, no pathogen effector has yet been identified with kinase activity toward InsPs, leaving a major gap in our mechanistic understanding of how these pathways might be subverted.

Phytase-type bacterial effectors such as XopH and AvrBs7, and fungal NUDIX hydrolases like *Mo*NUDIX, exemplify how different pathogen lineages converge on InsP metabolism as a means to promote host colonization. Whether these activities primarily promote virulence via suppression of immunity, increased phosphate release, induction of phosphate starvation responses to enhance phosphate uptake or mobilization from uninfected regions to the infected sites to support pathogen nutrition or operate through a combination of these mechanisms remains to be determined.

The convergence of InsP-targeting strategies across diverse kingdoms suggests that these metabolites represent central nodes of vulnerability within the plant’s immune-metabolic network. Going forward, elucidating the substrate specificity, structural basis, and *in vivo* consequences of effector–InsP interactions will be key to uncovering their roles in pathogenesis. Understanding these mechanisms will not only shed light on host–pathogen co-evolution but also guide the development of novel resistance strategies that safeguard InsP signaling pathways from microbial interference.
